# At the intersection of sexual and reproductive health and HIV services: use of moderately effective family planning among female sex workers in Kampala, Uganda

**DOI:** 10.1186/s12884-022-04977-5

**Published:** 2022-08-17

**Authors:** Avi J. Hakim, Moses Ogwal, Reena H. Doshi, Herbert Kiyingi, Enos Sande, David Serwadda, Geofrey Musinguzi, Jonathan Standish, Wolfgang Hladik

**Affiliations:** 1 Division of Global HIV and Tuberculosis, US Centers for Disease Control and Prevention, 1600 Clifton Rd, NE, US1-2, Atlanta, GA 30329 USA; 2 School of Public Health, Makerere University, Kampala, Uganda; 3 Division of Global HIV and Tuberculosis, US Centers for Disease Control and Prevention, Kampala, Uganda; 4 Department of Counseling and Psychological Services, Georgia State University, Atlanta, USA

**Keywords:** Female sex workers, Reproductive Health, HIV, Family planning, Uganda

## Abstract

**Background:**

Female sex workers are vulnerable to HIV, sexually transmitted diseases, and unintended pregnancies; however, the literature on female sex workers (FSW) focuses primarily on HIV and is limited regarding these other health issues.

**Methods:**

We conducted a respondent-driven sampling (RDS) survey during April-December 2012 to characterize the reproductive health of and access to contraceptives FSW in Kampala, Uganda. Eligibility criteria included age ≥ 15 years, residence in greater Kampala, and having sold sex to men in ≤ 6 months. Data were analyzed using RDS-Analyst. Survey logistic regression was used in SAS.

**Results:**

We enrolled 1,497 FSW with a median age of 27 years. Almost all FSW had been pregnant at least once. An estimated 33.8% of FSW were currently not using any form of family planning (FP) to prevent pregnancy; 52.7% used at least moderately effective FP. Among those using FP methods, injectable contraception was the most common form of FP used (55.4%), followed by condoms (19.7%), oral contraception (18.1%), and implants (3.7%). HIV prevalence was 31.4%, syphilis prevalence was 6.2%, and 89.8% had at least one symptom of a sexually transmitted disease in the last six months. Using at least a moderately effective method of FP was associated with accessing sexually transmitted disease treatment in a stigma-free environment in the last six months (aOR: 1.6, 95% CI: 1.1–2.4), giving birth to 2–3 children (aOR: 2.5, 95% CI: 1.4–4.8) or 4–5 children (aOR: 2.9, 95% CI: 1.4–5.9). It is plausible that those living with HIV are also less likely than those without it to be using a moderately effective method of FP (aOR: 0.7, 95% CI: 0.5–1.0).

**Conclusions:**

The provision of integrated HIV and sexual and reproductive health services in a non-stigmatizing environment has the potential to facilitate increased health service uptake by FSW and decrease missed opportunities for service provision.

## Background

The burden of HIV among female sex workers (FSW) has been well documented and data on progress toward the Joint United Nations Programme on HIV/AIDS (UNAIDS) 90–90-90 targets—90% of people living with HIV are diagnosed, 90% of them are on treatment, and 90% of them have suppressed viral load—are slowly becoming available [1–4]. FSW are 13.5 times as likely as women in the general population of lower and middle income countries to be infected with HIV and 5% of new HIV infections are among FSW in these countries [5]. Lower educational level, poverty, and mobility among FSW also may impact HIV acquisition or reduce health service uptake [6–9]. Missing, however, is an understanding of the sexual and reproductive lives of FSW, lives that are intricately connected with HIV risk and service uptake, but which are not integrated and instead addressed separately or not at all, both in the literature and in service delivery [10–13].

In addition to HIV, multiple sexual partners and inconsistent condom use make FSW vulnerable to other sexually transmitted diseases (STD) [5, 14]. These behaviors and low use of modern methods of family planning (FP) also make them vulnerable to unintended pregnancies [5, 14]. Research has shown that most FSW have had at least one pregnancy in their lifetime and more than half of FSW are estimated to have a curable STD at a given time [13, 15–17]. Pregnancy during sex work has also been associated with fewer live births and more terminated pregnancies [18].

The 2016 Uganda Demographic Health Survey (UDHS) found that 47% of sexually active unmarried women and 35% of married women used modern contraception (Macro International & Uganda Bureau of Statistics, 2018). Among sexually active unmarried women, injectables were the most common method (21%), followed by male condoms (14%). Nearly half (45%) of episodes of contraceptive use in the 5 years preceding the UDHS survey were discontinued within 12 months. Contraceptive discontinuation rates were highest for oral hormonal contraception (67%) and long-acting injectables (52%) and lowest for implants (21%). Discontinuation occurred most frequently due to health concerns or side effects. The survey also found that 41% of births or current pregnancies were mistimed or unwanted [19]. Consequently, the Ugandan Ministry of Health estimates that unsafe termination of pregnancies are responsible for 8% of maternal deaths [20].

A 2012 biobehavioral survey was conducted in Kampala, Uganda to characterize HIV prevalence and viral load suppression among FSW. The survey estimated HIV prevalence at 31.4% in this population. Among FSW living with HIV, 45.5% were aware of their infection [9]. Syphilis prevalence was 6.2% [9]. Sexually transmitted diseases are also common among FSW in Kampala. In 2008, approximately 22% had syphilis, 9% trichomonas vaginalis, 8% vaginal gonorrhea, 4% anorectal gonorrhea, 4% vaginal chlamydia, and 2% anorectal chlamydia [17, 21]. Here we examine uptake of reproductive health services among FSW in Kampala, Uganda, and factors associated with using at least a moderately effective method of FP defined here as including: oral hormonal contraception, long-acting injectables, implant, and intrauterine devices [22].

## Methods

Based on experience in a previous survey of FSW in Kampala, we utilized respondent-driven sampling (RDS) to recruit up to 1,500 FSW into the CRANE Survey between April and December 2012. The CRANE survey aimed to characterize HIV prevalence, risks behaviors, and health service utilization among FSW. RDS is a variant of snowball sampling that yields a probability-based sample [23–25]. Eligibility criteria included age 15 years or older, residence in greater Kampala, and having sold sex to men in the last six months. Recruitment began with four purposively selected seeds identified by survey staff. Seeds were selected to be diverse in terms of age, type of venue where they find clients (e.g., street, bar, hotel), and neighborhood where they find clients. At the first survey visit, participants were given the equivalent of $4 US in Ugandan Shillings for their time and transportation. Those who successfully recruited peers who participated in the survey prior to the second visit received an additional amount in Ugandan Shillings equivalent to $1.25 US per recruit. Participants could recruit a maximum of three people. This was reduced gradually to two, one, and finally zero as the desired sample size was attained. Detailed methods have been described elsewhere [9].

### Data collection

After eligibility screening and the provision of verbal informed consent, participants took an audio-computer assisted self-interview in English or Luganda. Interview domains included demographics, sexual history, condom and lubricant use, reproductive health, sexually transmitted diseases, and uptake of health services. Healthcare associated stigma was determined by asking participants who accessed treatment for a sexually transmitted disease in the 12 months if they felt stigmatized by healthcare staff. All results save for syphilis prevalence are self-reported.

Upon completion of the interview, participants received HIV pre-test counseling followed by blood draw for HIV testing as described elsewhere [9]. Plasma was also tested for syphilis using the anti-syphilis IgG ELISA (Biotec Laboratories, Suffolk, UK) and, if reactive, the rapid plasma reagin Syfacard-R Test (Murex Biotech, Dartford, UK). Participants with rapid plasma reagin-reactive test results were classified as having active syphilis infection.

Test results were returned to participants at the second survey visit which occurred approximately three weeks after the first. Those testing positive for HIV were referred to care and treatment. Those testing positive for syphilis were offered treatment at the survey site.

### Data measures and analysis

The variable of interest and dependent variable for logistic regression *was use of at least moderately effective FP* defined here as including: oral hormonal contraception, long-acting injectables, implant, and intrauterine devices. Data were analyzed in RDS-Analyst (Los Angeles, CA) version 5.7 using Gile’s Successive Sampling estimator to develop weighted population estimates and 95% confidence intervals. Weighted logistic regression was conducted in SAS (SAS Institute Inc., Cary, NC) using survey logistic procedures to identify correlates of use of at least a moderately effective form of FP. Missing data were treated as such and not included in any analyses. Variables were considered for inclusion in the model based on the published literature, and those significant at the 0.1 level in the bivariate analysis were included in the multivariable analysis. Adjusted odds ratios (aOR) and their 95% confidence intervals (CI) are presented.

### Ethics approvals

This survey was approved by the ethical review boards of Makerere University School of Public Health and the Uganda National Council of Science and Technology, as well as the Centers for Disease Control and Prevention as a research activity involving human subjects. We obtained verbal informed consent from participants to participate in the survey. The use of verbal informed consent was approved because written consent would be the only identifiable information collected and could pose a risk to participants. A waiver to obtain informed consent from parents or guardians of participants under the age of 18 was granted as the risks of participation were minimal and outweighed by the potential risks of disclosure of sex work to parents or guardians. No personal identifiers were collected. All methods were performed in accordance with the relevant guidelines and regulations.

## Results

We enrolled and analyzed data from 1,497 FSW. The median age of FSW in Kampala was 27 years and 32.7% were between the ages of 15 and 24 years (Table 1). Almost half (49.5%) had never been married. And 59.6% had at least one steady sex partner in the last six months (data not shown). One-fifth (22.2%) had sold sex for less than one year and 35.6% for 1–2 years.

**Table 1:** Demographic characteristics for female sex workers, crude and weighted results, Crane Survey, Kampala, Uganda, 2012

Characteristic	n (*N* = 1,497)	Sample Proportion (%)	Population Proportion % (95% CI)
Age, in years (median, IQR)	28 (23–32)		27 (23–32)
15–24	480	32.1	32.7 (29.4–35.9)
25–34	759	50.7	50.0 (26.9–53.1)
35–49	258	17.2	17.3 (14.9–19.7)
Religion			
Protestant	433	29.2	29.4 (25.9–32.9)
Catholic	543	36.6	34.3 (30.7–37.9)
Muslim	412	27.8	29.1 (25.7–32.5)
Other	83	5.6	6.0 (4.1–7.8)
None	13	0.9	1.2 (0.4–2.0)
Years of schooling (median, IQR)	6 (0–10)		6 (0–9)
None	539	36.3	37.6 (34.6–40.6)
1–7	458	30.8	30.6 (27.9–33.4)
8–13	357	24.0	23.3 (20.6–25.9)
≥ 14	133	8.9	8.5 (6.7–10.2)
Current marital status			
Never married	747	50.2	49.5 (46.5–52.4)
Married	91	6.1	5.9 (4.6–7.3)
Divorced	272	18.3	19.0 (16.6–21.4)
Separated	302	20.3	20.2 (17.8–22.7)
Widow	75	5.0	5.3 (3.9–6.7)
Age at initiation of sex work			
< 25	692	46.6	45.3 (42.1–48.5)
≥ 25	792	53.4	54.7 (51.5–57.9)
Years in sex work (median, IQR)	2 (1–5)		2 (1–4)
< 1	302	20.3	22.2 (19.5–24.9)
1–2	483	32.5	35.6 (32.6–38.7)
3–5	439	29.5	26.5 (24.0–29.1)
≥ 6	263	17.7	15.6 (13.5–17.8)
Sex work as main source of income			
Yes	1401	94.2	94.4 (93.0–95.9)
No	86	5.8	5.6 (4.1–7.0)

*IQR* Interquartile range, *CI* Confidence intervals

Table 2 shows sexual behaviors and reproductive health characteristics among Kampala FSW. Less than 1 in 10 FSW (9.5%) had anal sex in their lifetime. Approximately two-thirds (65.2%) of FSW used a condom at last sex. Pregnancy history was common among FSW, with 88.6% having been pregnant and 8.6% currently pregnant. Over one-quarter (27.2%) had given birth to at least four children. A similar proportion (29.9%) had had at least one miscarriage and 37.4% had terminated one or more pregnancies. An estimated 35.3% were not using use any form of FP, including condoms, to prevent pregnancy, and 19.4% did not have easy access to FP services. Roughly equal proportions of those who were pregnant and those who were not pregnant did not have easy access to FP (data not shown). Among FSW using FP, a variety of methods were used, including injectables (55.4%), oral contraception (18.1%), implants (3.7%), and intrauterine devices (2.4%). Condoms were relied upon by 19.7% of FSW using a FP method.

**Table 2:** Sexual behaviors and reproductive health among female sex workers; Crane Survey, Kampala, Uganda, 2012

**Characteristic**	**n** (*N* = 1,497)	**Sample Proportion (%)**	**Population Proportion** **% (95% CI)**
Type of sex engaged in, ever			
Vaginal	1333	90.5	90.5 (88.7–92.4)
Anal	21	1.4	1.8 (0.9–2.8)
Both	119	8.1	7.7 (6.1–9.3)
Condom use at last sex, any partner			
Yes	302	66.8	65.2 (60.0–70.5)
No	150	33.2	34.8 (29.5–40.0)
Currently pregnant			
Yes	112	8.6	8.6 (6.9–10.2)
No	1195	91.4	91.4 (89.8–93.1)
Ever been pregnant			
Yes	1307	88.7	88.6 (86.7–90.6)
No	166	11.3	11.4 (9.4–13.4)
Number of children given birth to			
0	203	13.8	14.1 (11.8–16.4)
1	236	16.0	15.4 (13.4–17.5)
2–3	616	41.8	43.3 (40.3–46.3)
4–5	308	20.9	19.9 (17.6–22.3)
6 +	110	7.5	7.3 (5.7–8.9)
Number of miscarriages, ever			
0	905	70.5	70.1 (67.0–73.1)
1	275	21.4	21.1 (18.4–23.8)
2	103	8.0	8.8 (6.9–10.8
Number of pregnancies terminated, ever			
0	823	63.0	62.6 (59.4–65.8)
1	241	18.4	20.3 (17.1–22.2)
2	169	12.9	11.7 (9.7–13.8)
3 +	74	5.7	5.4 (4.0–6.8)
Can easily get family planning services, currently			
Yes	1187	80.6	80.6 (78.1–83.1)
No	286	19.4	19.4 (16.9–21.9)
Among those not pregnant, currently using any family planning methods			
Yes	924	65.9	66.2 (63.2–69.1)
No	437	34.1	33.8 (30.9–36.8)
Among those using family planning, method used			
Oral contraception	161	17.9	18.1 (15.0–21.3)
Hormonal injection	497	55.5	55.4 (51.5–59.4)
Implant	35	3.8	3.7 (2.4–5.0)
Intrauterine device	18	1.5	2.4 (0.9–3.9)
Condoms	207	20.7	19.7 (16.7–22.4)
Other	6	0.6	0.7 (0.1–1.3)
Among those not pregnant, used at least a moderately effective method of family planning^a^			
Yes	711	52.2	52.7 (49.7–55.8)
No	650	47.8	47.3 (44.3–50.3)

*CI* Confidence intervals

^a^ Moderately effective method of FP defined here as including: oral hormonal contraception, long-acting injectables, implant, and intrauterine devices

While lubricants were used by 36.0% of FSW, 52.4% of those who used lubricants used oil-based lubricants (Table 3). Symptoms of sexually transmitted diseases were common, with 89.8% of FSW reporting at least one in the last six months (Table 3). Of those with symptoms, 63.0% reportedly had lesions or ulcers and 22.5% reported warts (data not shown). Though 68.5% of FSW with STD symptoms sought treatment from a hospital, clinic, or pharmacy, 31.4% either self-treated or did not access treatment at all. Among those who sought treatment from a hospital, clinic, or pharmacy, 35.9% felt stigmatized by healthcare provider. Meanwhile, 17.1% of FSW felt they did not have easy access to STD treatment. Just over half of FSW with STD symptoms (53.6%) stopped having sex when they had an STD.

**Table 3:** Utilization of condoms, lubricants, and STD services among female sex workers; Crane Survey, Kampala, Uganda, 2012

**Characteristic**	**n** (*N* = 1,497)	**Sample Proportion (%)**	**Population Proportion** **% (95% CI)**
Run short of condoms in last six months			
Yes	207	45.8	47.5 (41.8–53.3)
No	245	54.2	52.5 (46.7–58.2)
Reason run short of condoms			
Not available	140	67.6	65.7 (58.6–72.4)
Embarrassed to buy	35	16.9	18.8 (13.1–24.7)
Too expensive	26	12.6	13.1 (8.2–17.9)
Other	6	2.9	2.5 (0.1–4.8)
Ever used female condoms			
Yes	101	22.4	21.0 (15.9–25.9)
No	349	77.6	79.0 (74.1–84.1)
Ever used lubricant during sex			
Yes	550	37.0	36.0 (33.1–38.9)
No	936	63.0	64.0 (61.2–66.9)
Type of lubricant used			
Water-based	244	47.7	47.6 (41.6–51.6)
Oil-based	267	52.3	52.4 (45.4–55.4)
Ever re-use condoms			
Yes	69	15.3	14.2 (10.4–17.9)
No	383	84.7	85.8 (82.2–89.6)
Ever tested for HIV			
Yes	1069	71.9	71.9 (69.0–74.9)
No	418	28.1	28.1 (25.3–30.9)
Had STD symptoms in last six months			
Yes	1338	89.4	89.8 (87.9–91.6)
No	159	10.6	10.3 (8.4–12.1)
Stopped having sex during symptoms			
Yes	622	52.8	53.6 (50.2–57.0)
No	555	47.2	46.4 (43.0–49.8)
Among those with STD symptoms, location of STD treatment			
Hospital or clinic	581	49.5	48.6 (45.2–52.1)
Pharmacy	225	19.2	19.9 (17.1–22.7)
Treated myself	184	15.7	15.7 (13.3–18.0)
Did not treat	183	15.6	15.7 (13.3–18.1)
Have easy access to STD treatment			
Yes	1218	81.9	82.9 (80.8–85.1)
No	269	18.1	17.1 (14.9–19.2)
Experienced stigma from healthcare worker when obtaining STD treatment			
Yes	274	34.0	35.9 (31.8–40.1)
No	532	66.0	64.1 (59.9–68.2)

*CI* Confidence intervals

In multivariable analysis (Table 4), the odds of using at least a moderately effective method of FP was higher among women who had not experienced stigma from a healthcare worker when obtaining STD treatment in the last six months compared to those who had (aOR: 1.6, 95% CI: 1.1–2.4) and 2. Women who have given birth to 2–3 children (aOR: 2.5, 95% CI: 1.4–4.8) and 4–5 children (aOR: 2.9, 95% CI: 1.4–5.9) It is plausible that those living with HIV are also less likely than those without it to be using a moderately effective method of FP (aOR: 0.7, 95% CI: 0.5–1.0).

**Table 4:** Multivariable analysis on factors correlated with using at least a moderately effective method of family planning; Crane Survey, Kampala, Uganda, 2012

**Characteristic**	**Unadjusted**		**Adjusted**	
	**OR (95% CI)**	***p*-value**	**aOR (95% CI)**	***p*-value**
Age				
15–24	Ref	0.016	Ref	0.728
25–34	1.5 (1.1–2.0)		1.1 (0.6–2.0)	
> 35	1.0 (0.7–1.4)		0.9 (0.6–1.5)	
Marital status				
Never married	Ref	0.019	Ref	0.595
Married	1.5 (0.9–2.5)		1.3 (0.7–2.7)	
Divorced or separated	1.5 (1.2–1.9)		1.1 (0.7–1.6)	
Widowed	0.9 (0.5–1.7)		0.7 (0.3–1.6)	
Years of schooling				
None	Ref	0.707	-	
1–7	0.9 (0.6–1.2)			
8–13	0.8 (0.6–1.2)			
> 14	0.8 (0.5–1.3)			
Years in sex work				
< 1	Ref	0.972	-	
1–2	1.1 (0.7–1.6)			
3–5	1.1 (0.7–1.5)			
> 6	1.1 (0.7–1.6)			
Number of children given birth to				
0	Ref	< .0001	Ref	0.011
1	2.2 (1.3–3.6)		1.8 (0.9–3.6)	
2–3	3.1 (2.0–4.6)		2.5 (1.4–4.8)	
4–5	3.2 (2.0–5.2)		2.9 (1.4–5.9)	
6 +	2.4 (1.3–4.6)		1.3 (0.6–3.2)	
Number of pregnancies terminated				
0	Ref	0.541	-	
1	1.2 (0.8–1.8)			
2 +	1.1(0.7–1.7)			
3 +	1.4(0.8–2.6)			
Experienced stigma from healthcare worker when obtaining STD treatment				
Yes	Ref	0.035	Ref	0.011
No	1.5 (1.1–2.1)		1.6 (1.1–2.4)	
Ever tested for HIV				
Yes	Ref	0.004	Ref	0.624
No	0.7 (0.5–0.9)		1.1 (0.7–1.7)	
HIV status				
Negative	Ref	0.047	Ref	0.054
Positive	0.8 (0.6–1.0)		0.7 (0.5–1.0)	
Syphilis status				
Negative	Ref	0.832	-	
Positive	0.9 (0.6–1.6)			

*OR* Odds ratio, *aOR* Adjusted odds ratio, *CI* Confidence intervals

## Discussion

Approximately half (52.7%) FSW in Kampala used at least a moderately effective method of family planning [26]. Barriers to FP are many and may include an unsupportive health clinic environment, including inconvenient hours and discriminatory providers as key barriers to contraceptive use [27]. We found that 35.9% of FSW with an STD felt stigmatized by healthcare workers when seeking STD treatment. Sex worker experiences of stigmatization by healthcare providers have been well documented but little data exist reflecting its impact on healthcare utilization [28–31]. We found that for FSW in Kampala, increased odds of using at least a moderately effective method of FP was associated with stigma-free STD services. Key population sensitization training for healthcare providers can facilitate the expansion of stigma-free services for FSW therefore has the potential to increase utilization of moderately effective FP methods. It is plausible that the use of moderately effective FP was also inversely associated with living with HIV, suggesting that there may not be integration of HIV and reproductive health services. This has important clinical and public health implications, particularly if those living with HIV have not attained viral suppression.

Sexually transmitted diseases are a risk factor for HIV acquisition and transmission [32, 33]. They are also a public health problem in their own right, particularly for a population such as FSW with high prevalence of STDs such as syphilis that can affect birth outcomes [34, 35]. Nearly nine in ten experienced STD symptoms in the last six months. Approximately two-thirds (68.5%) of those with STD symptoms sought treatment at a hospital, clinic, or pharmacy. In contrast, though approximately one-third of FSW in Kampala were living with HIV, 28.1% of FSW had never tested for HIV and of those who have, 67.6% did so in the last 12 months, and self-reported awareness of living with HIV was 45.5% [9]. Though STD prevalence is lower than HIV prevalence among FSW in Kampala, FSW seem more inclined to seek care for STD symptoms than they do testing for HIV, perhaps because they experienced symptoms that needed to be addressed.

Many FSW are more interested in testing and treatment for STD than for HIV, possibly because until the late stages of the disease HIV may go unnoticed, whereas STD symptoms cause discomfort, may be more obvious to others, and may negatively impact their ability to earn money [29]. Furthermore, most STD can be cured, usually with one to three clinic visits [34–36]. STD screening, testing, and treatment provision can potentially attract women to health services where they may also be offered pregnancy testing and linkage to maternity care if pregnant, FP and HIV testing. STD services consequently are an opportunity not only for FP and HIV testing, but to inform women about the benefits of these services, for themselves and others, including children they may have in the future. For such integrated services to be successful, it is imperative that they be provided in a stigma-free environment.

In 2008, the median duration of sex work among current FSW was three years [17]. By 2012, the median duration was two years, suggesting that women may be remaining in sex work for less time. This scenario of a higher turnover rate could lend itself to unchanged HIV and STD incidence among FSW as those entering the profession would be less likely to already be infected than those who have been in it for longer. This is supported by the unchanged HIV prevalence among FSW in Kampala between 2008 and 2012 [17]. And as these FSW become infected with HIV and remain undiagnosed and not on antiretroviral treatment (ART), population viral load will remain elevated and consequently so will the potential for transmission to clients and then from clients to other sex workers and the general population.

Although abortion is illegal in Uganda, 37.4% of FSW have terminated at least one pregnancy in their lifetime, similar to other locations in sub-Saharan Africa [37]. Unable to obtain an abortion from trained healthcare providers, FSW may terminate their pregnancies on their own or with the assistance of unskilled individuals, thereby increasing the risk of maternal morbidity and mortality [37–39]. It also points to the potential unmet need for effective methods of FP.

Our findings are limited by the cross-sectional nature of our survey and that we did not ask participants if they obtain HIV, STI, and FP services from a single integrated site or separate sites, though at the time of data collection FSW service providers were focused primarily on HIV. We also did not ask women if they were trying to avoid pregnancy and about uptake of prevention of mother-to-child transmission of HIV (PMTCT) services. As FSW programs in Uganda do not provide PMTCT services, FSW would need to access these at general population clinics where they may receive multi-layered stigma derived from the combination of factors (e.g., selling sex while pregnant, being HIV positive and pregnant, being an HIV-positive sex worker). FSW, therefore, may encounter negative attitudes from multiple sources when seeking PMTCT services—service providers, other sex workers, other women accessing health services, and community members [31, 40–42]. Additionally, our analysis may overestimate associations with using at least a moderately effective method of family planning because using such a method was relatively common. The age of these data highlight the infrequency with which biobehavioral surveys of female sex workers are conducted.

There are many opportunities for integration of sexual and reproductive health services with HIV services. In Kampala and elsewhere, a sizeable share of FSW experiencing STD symptoms obtain treatment directly from a pharmacy. Pharmacists can be trained to offer contraceptives, HIV testing, or HIV self-tests along with referrals to FSW-friendly health services [43, 44]. Similarly, drop-in center staff and outreach workers can similarly be trained to provide such services. As FSW on ART are already engaged with the healthcare system, every reproductive or other health service not provided to them during ART visits is a missed opportunity. Among FSW on ART in Kampala, 8.2% had active syphilis infection [9, 45]. For these women, each visit with an HIV treatment provider can be an opportunity to screen for and treat STD and discuss FP.

## Data Availability

As the study population is stigmatized and vulnerable, the datasets analyzed during the current study are available from the corresponding author on reasonable request.
